# Determinants of favorable or unfavorable opinion about euthanasia in a sample of French cancer patients receiving palliative care

**DOI:** 10.1186/s12904-018-0357-6

**Published:** 2018-08-29

**Authors:** Alexandre de Nonneville, Théo Chabal, Anthony Marin, Jean Marc La Piana, Marie Fichaux, Véronique Tuzzolino, Florence Duffaud, Pascal Auquier, Augustin Boulanger, Karine Baumstark, Sébastien Salas

**Affiliations:** 1 Aix Marseille University, 13005 Marseille, France; 2 Department of Oncology and Palliative Care, Timone Hospital, 13005 Marseille, France; 3 Département de médecine générale, Aix Marseille Université, Marseille, France; 4 La Maison, Gardanne, France; 5 Unité d’Aide Méthodologique à la Recherche Clinique et Epidémiologique, AP-HM, Marseille, France; 6 Self-perceived Health Assessment Research Unit, Aix Marseille Université, EA3279, Marseille, France; 7 Department of Oncology and palliative care, Hôpital de la Timone, 264 Rue Saint-Pierre, 13385 Marseille, France

**Keywords:** Euthanasia, Opinions, Cancer, Patients, Palliative care

## Abstract

**Background:**

Opinion about euthanasia has been explored among the general population and recently in patients receiving palliative care. 96% of the French population declared themselves in favor of euthanasia while less of 50% of palliative care patients are. The aim of the present study was to explore and identify potential determinant factors associated with favorable or unfavorable opinion about euthanasia in a French population of cancer patients receiving palliative care.

**Methods:**

We performed a cross-sectional study among patients in two palliative care units. Eligible patients were identified by the medical staff. Face-to-face interviews were performed by two investigators. Two groups were defined as favorable or unfavorable about euthanasia according to the answer on the specific question about patient opinion on euthanasia. A multivariate analysis including age, belief in God, chemotherapy and gender was built.

**Results:**

Seventy-eight patients were interviewed. Median age was 60.5 years (range: 31–87.2). In univariate analysis, patients with a favorable opinion were most often under 60 years old (62 versus 38% unfavorable; *p = 0.035*), in couple (64 versus 35%; *p = 0.032*), didn’t believe in God (72 versus 28% were non-believers; *p < 0.001*) and had more frequently an history of chemotherapy treatment (58 versus 42% received at least one cycle of chemotherapy; *p = 0.005*). In a multivariate analysis, age <  60 years, absence of belief in God and an antecedent of chemotherapy were independently associated with a favorable opinion about euthanasia (OR = 0.237 [0.076–0.746]; *p = 0.014*, OR = 0.143 [0.044–0.469]; *p = 0.001, and* OR = 10.418 [2.093–51.853]; *p = 0.004,* respectively).

**Conclusion:**

We report here determinants of opinion about euthanasia in palliative care cancer patients. Thus, young patients who do not believe in God and have a history of chemotherapy treatment are more likely to request the discontinuation or restriction of their treatment. A better understanding of these determinants is essential for the development of information and/or interventions tailored to the palliative context.

**Electronic supplementary material:**

The online version of this article (10.1186/s12904-018-0357-6) contains supplementary material, which is available to authorized users.

## Background

Societal issues related to end of life and euthanasia have led to substantial public debate worldwide [1]. In Europe, euthanasia legislation differs from one country to another and is legalized in only three countries [2]. Because of many factors of social, cultural, historical or religious nature, even the definition of euthanasia could be controversial. Based on French law, euthanasia can be defined as “the act of a third party who deliberately ends a person’s life with the intention of putting an end to a situation deemed unbearable” (French National Consultative Committee on Ethics, CCNE, Opinion No. 63, January 2000). In France, the question whether euthanasia should be legalized has been topical for several years. A first law regulated patients’ rights and end-of-life care, called the Leonetti Law [3], after the man who originally proposed the law, in 2005. This law explicitly allows physicians to provide far-reaching symptom control, even at the risk of shortening life, in order to relieve the person’s suffering at an advanced stage of a serious and incurable disease. Thus the concept of double effect was introduced, while prohibiting physician-assisted suicide and euthanasia. It has been suggested that the Leonetti’s law also allows continuous deep sedation as a form of ongoing symptom control [4], although it does not mention the practice. The end of life remains a topic of legal, political and ethical debate in France. After the re-examination by a parliamentary commission of questions related to the accompaniment of patients at the end of life and euthanasia, the French Government voted a new law in February 2016 called the Claeys-Leonetti Law [5], which introduce various significant amendments to the existing Leonetti’s law. This law establishes the right to deep and continuous sedation, consisting of sedative and analgesic treatment leading to a profound and continuous change of vigilance to death if the patient is likely to suffer pain, associated with the cessation of all life-sustaining treatments including artificial nutrition and hydration [6]. Claeys-Leonetti law increased patients’ autonomy by strengthening the value of advance directives and extending the spectrum of unreasonable obstinacy to the sustainment of vital treatments [7]. The law also confirmed the ban on euthanasia per se [5]. However, while 96% of French people have been found to be in favor of euthanasia [8], fewer than 50% of physicians are [9]. We recently explored opinion about euthanasia among patients receiving palliative care [10]. This previous study showed that patients in an end-of-life setting are probably more reticent to legalize euthanasia than general population since only 48% of palliative care patients were favorable to this practice. This reflects a large difference of opinion between the general population, healthy, and patients with a fatal incurable disease such as cancer. The aim of the present study was to explore and identify potential determinants factors associated with favorable or unfavorable opinion about euthanasia in a French population of cancer patients in palliative care units.

## Methods

### Design and setting

We performed a cross-sectional study among patients in two palliative care units: Timone University Hospital (Marseille, France) and La Maison (Gardanne, France, non-profit association under the terms of the french 1901 law), which is an institution run by a non-profit association providing palliative care. Eligible patients were identified by the medical staff. They were selected after an initial interview with psychologists and a members of the medical team in order to identify anxious or depressed patients and those who would not be physically and/or psychologically able to answer questions during the study interview. Two investigators conduct face-to-face interviews on the two sites alternatively. A translation of the interview questionnaire is available in Additional file 1. Before starting the interview, investigators presented the purpose of the study and the nature of the questions to the patient. The interview did not last longer than 30 min.

### Population

Inclusion criteria were as follows: age ≥ 18 years old; locally advanced or metastatic cancer; patient with palliative care (as defined by the French Society of Palliative and Support Care, SFAP, as active care in a global approach to persons with progressive or terminal illness) [6] in a palliative care unit or in specific palliative care beds in non-palliative care units; without sleepiness (Epworth scale: score > 16) [11]; without anxiety and/or mood disorder (HAD scale: score < 7) [12]; and providing written informed consent for participation. Patients already sedated or those unable to communicate or to understand the purpose and conditions of the study (based on the clinician’s judgment) were excluded.

### Data collection

Socio-demographic data (age, gender, educational level, professional activity, family situation) and clinical data (chemotherapy yes/no, regardless the number of line or cycle of treatment) were collected from medical records then confirmed during the interview. Pain data was collected during interview (visual analogic pain scale from 0 to 10; 0 no pain, 10 maximal pain expressed as a categorical variable: yes (pain > 3/10) / no (pain ≤3/10) and paroxysmal pain (maximal pain they had felt during their hospitalization) [13]; mention of believing in God (yes/no); a question about the quality of global information they received about their treatments (well-informed: yes/no); a question about support from family and friends (yes/no); a question about whether they had given advance directives (yes/no) and a specific question (yes/no) about their opinion on euthanasia.

### Ethics, consent and permission

All procedures performed in this study involving human participants were done in accordance with the French ethical standards and with the 2008 Helsinki declaration. As this was a non-interventional study, ethical approval was not needed, according to French law (Article L1121–1, Law n°2011–2012 29 December 2011 - art. 5). All subjects participated on a voluntary basis. Written consent for participation in the study was obtained from all participants.

### Statistical analysis

Categorical variables were described using counts and frequencies and quantitative variables were described using medians and ranges. Two groups were defined as favorable or unfavorable about euthanasia according to the answer on the specific question about patient opinion on euthanasia. Patients’ characteristics were compared with χ2 or exact Fisher’s tests for discrete variables and rank-Wilcoxon’s tests for continuous variables according patient opinion on euthanasia. A multivariate analysis was conducted using logistic regression including variables selected on their clinical interest and/or a threshold *p*-value ≤0.05 during univariate analysis: age (≤60 vs. > 60 years), belief in God (no vs. yes), chemotherapy (no vs. yes) and gender (female vs. male). Results were expressed as odds ratios and their 95% confidence intervals. The level of statistical significance was set at 1-α = 0.95. Statistical analyses were carried out with the SPSS® software version 17.

## Results

### Patients

A total of 78 patients were interviewed. Median age was 60.5 years (range 31 to 87.2), 56% were women (*n* = 44), 40% were in a couple (*n* = 31) and 54% believed in God (*n* = 42). The main cancer sites were pulmonary (24%; *n* = 19), digestive (20%; *n* = 16), head and neck (14%; *n* = 11) and gynecologic (13%; *n* = 10). 79% had already received chemotherapy (*n* = 62).

### Opinion about euthanasia

50% of patients interviewed had a favorable opinion about euthanasia (*n* = 39).Univariate analysis (Table 1) showed differences between patients with a favorable opinion and those without. Patients with a favorable opinion were most often under 60 years old (62% of patients < 60 years old were favorable versus 38% unfavorable; *p = 0.035*), in couple (64 versus 35%; *p = 0.032*), didn’t believe in God (72 versus 28% were non-believers; *p < 0.001*) and had more frequently an history of chemotherapy treatment (58 versus 42% received at least one cycle of chemotherapy; *p = 0.005*). Pain level did not statistically impact the opinion about euthanasia despite a tendency, patients with a visual analogic pain scale > 3 seemed less favorable to euthanasia (*p = 0.080*). Gender, educational degree, information on treatment and redaction of advance directives were not statistically associated with patients’ opinions. In a multivariate analysis (Table 2), age <  60 years and absence of belief in God were independently associated with a favorable opinion about euthanasia (OR = 0.237 [0.076–0.746]; *p = 0.014* and OR = 0.143 [0.044–0.469]; *p = 0.001* respectively). Absence of previous use of cytotoxic chemotherapy was independently associated with an unfavorable opinion about euthanasia (OR = 10.418 [2.093–51.853]; *p = 0.004*).

**Table 1:** Patients characteristics and univariate analysis between individuals with a favourable opinion about euthanasia and those without. %, percentages are calculated in relation to the number of available data

		Favourable opinion about euthanasia		
	All	No	Yes	*p-value* *(Khi* ^*2*^ *or Fisher)*
Age				
Median	60.5 [31–87]	64 [31–87]	58.8 [45–78]	
< 60y	39 (50)	15 (38.5)	24 (61.5)	0.035
≥ 60y	39 (50)	24 (61.5)	15 (38.5)	
Sex				
Male	34 (43.6)	14 (41.2)	20 (58.8)	0.127
Female	44 (56.4)	25 (56.8)	19 (43.2)	
Educational degree				
No diploma	43 (55.1)	25 (58.1)	18 (41.9)	0.271
Graduate	19 (24.4)	8 (42.1)	11 (57.9)	
Post graduate	16 (20.5)	6 (37.5)	10 (62.5)	
Professional activity				
No	39 (50)	20 (51.3)	19 (48.7)	0.500
Yes	39 (50)	19 (48.7)	20 (51.3)	
Family situation				
Single	47 (60.3)	28 (59.6)	19 (40.4)	0.032
In couple	31 (39.7)	11 (35.5)	20 (64.5)	
Belief in God				
No	36 (46.2)	10 (27.8)	26 (72.2)	0.000
Yes	42 (53.8)	29 (69.0)	13 (31.0)	
Chemotherapy				
No	16 (20.5)	13 (81.3)	3 (18.8)	0.005
Yes	62 (79.5)	26 (41.9)	36 (58.1)	
Regular information on treatments				
No	23 (29.5)	9 (39.1)	14 (60.9)	0.160
Yes	55 (70.5)	30 (54.5)	25 (45.5)	
Pain				
No	49 (62.8)	21 (42.9)	28 (57.1)	0.080
Yes	29 (37.2)	18 (62.1)	11 (37.9)	
Paroxysmal pain				
No	9 (11.5)	5 (55.6)	4 (44.4)	0.500
Yes	69 (88.5)	34 (49.3)	35 (50.7)	
Surrounded by family				
No	14 (17.9)	9 (64.3)	5 (35.7)	0.188
Yes	64 (82.1)	30 (46.9)	34 (53.1)	
Advance directives done				
No	71 (91)	37 (52.1)	34 (47.9)	0.215
Yes	7 (9)	2 (28.6)	5 (71.4)

**Table 2:** Multivariate analysis including variables selected on their clinical interest and/or a threshold *p*-value ≤0.05 during univariate analysis: age (≤ 60 or > 60 years), belief in God, chemotherapy and sex

*Test*	*Contrast*	*Odd Ratio [CI 95%]*	*p-value*
Age	< 60 vs. ≥ 60 years	0.237 [0.076–0.746]	0.014
Belief in God	No vs. Yes	0.143 [0.044–0.469]	0.001
Chemotherapy	No vs. Yes	10.418 [2.093–51.853]	0.004
Sex	Female vs. Male	0.997 [0.317–3.130]	0.996

## Discussion

While 96% of French people have been found to be in favor of euthanasia, our previous study showed that palliative care patients, like physicians, are more reluctant to legalize euthanasia than the general population [8]. To further understand this area, factors associated with opinions regarding euthanasia seems to be a key issue. Other authors have instigated attitudes of patients with advanced cancer towards euthanasia and/or living wills but did not report any determinants factors associated with favorable or unfavorable opinion [14–16]. To the best of our knowledge, our study is the first identifying determinants associated with favorable or unfavorable opinion about euthanasia in cancer patients receiving palliative care. Having a favorable opinion about euthanasia was independently associated with an age under 60 years, the lack of belief in God and an antecedent of chemotherapy treatment. The impact of young age is in contradiction with the data of De Vleminck A et al. [17] in general population. In a Belgian cohort, they report an association between age > 55 years and making advance directives about euthanasia. Moreover, the euthanasia registration documents compiled between 1 January 2008 and 31 December 2009 and examined by the Federal Commission for the Control and Evaluation of Euthanasia in Belgium showed that half of the acts of euthanasia performed concerned patients between 60 and 79 years old [18]. Religions may regard understanding death and dying as vital to finding meaning in human life [19]. So it’s not surprising that all faiths have strong views on euthanasia and that believer are more reluctant to it. Indeed, the independent effect of religiosity has been extensively discussed in previous studies in general population [20, 21]. Other study showed that stronger religious belief was associated with more positive support for euthanasia [22]. However, the measures used to assess religiosity in these studies were not the same and again, general population was considered. Nevertheless, “to believe in God” is a rather simplistic way to explore religiosity and spirituality and their impact upon wish to hasten death and euthanasia. Some authors built a religiosity index including different items [23]. Other studies used more specific questions as the “religious affiliation” [24], the “belief in the church’s teachings” [25], or the “attendance to religious services” [26]. The third independent factor associated with a favorable opinion was the antecedent of cytotoxic chemotherapy treatment. Unfortunately, we did not have access to data on tolerance and toxicity assessments during the cytotoxic treatment due to the multiple centers involved in the treatment of patients prior to their arrival in the palliative care unit. This information could have helped in the interpretation of this data. However, we can make some assumptions. The succession of drug treatments of these patients may have trivialized the interventionism of doctors and cause patient lassitude. Moreover the patient has been confronted with announcements of therapeutic failures thereby placing him in a position where he may feel neglected, Unlike patients for whom exclusive palliative care has been undertaken from the outset and would be well perceived as active.

Family situation had a statistical impact on opinion about euthanasia only in univariate analysis. An explanation for this may be that patients living in a couple might not want to be a burden for their partner by undergoing protracted agony. However, another explanation may be found in a study of the Austrian population in which W.J. Stronegger [27] found less rejection of active euthanasia among couples and people with children. Another finding was that information given by medical staff about the treatment did not influence our patients’ opinion about euthanasia. This could be a bias because most of patients in palliative care are generally well informed about their treatment, its side-effects and the possibility of improving the last moments of their life. In addition, educational level is probably responsible for a different representation of euthanasia but did not influence significantly our patients’ opinion about it. Furthermore, level of pain was not associated with adhesion to euthanasia. This finding is in contradiction to another study that found, albeit in a very small number of patients, one of the principal reasons for requesting euthanasia was fear of future pain [28].

Our study has some limitations, lying mainly in the sample size and in the simplification of the questionnaire imposed by the context of patient vulnerability at the end of life. We arbitrary decided to consider the euthanasia opinion as a binary variable. Amount of information that patients wanted to share with us could be impacted by the idea they had about our own point of view on euthanasia. However, this approach was already used in similar works [26, 29] and leads to easier interpretation and presentation of results. Although investigating a wish to hasten death and opinions regarding euthanasia is of interest to for the development of information and/or interventions tailored to the palliative context, it poses considerable difficulties in terms of research. It raises questions about our current ability to care for and accompany patients through this most difficult of life stages. Medical advances, which have transformed diseases that once led to a quick death into chronic illnesses, coupled with increased life expectancy and other social phenomena linked to development, make it likely that far from being eradicated these situations will become more common [30]. The main contribution of this study is to demonstrate the feasibility of broaching sensitive issues such as euthanasia and cessation of care with patients in vulnerable situations. Based on this, we are currently conducting a national multicenter prospective study aimed to interview a thousand patients about the various points covered in the Cleays-Leonetti law, in order to minimize biases but also exploring a greater number of variables. We hope that our findings will be taken into account by lawmakers in France in order to satisfy the wishes of patients as well as possible.

## Conclusions

The present study reports determinants of opinion about euthanasia in palliative care cancer patients. Thus, young patients who do not believe in God and who have a history of chemotherapy are more likely to request the discontinuation or restriction of their treatment. A better understanding of these determinants is essential for the development of information and/or interventions tailored to the palliative context.

## Additional file

Additional file 1: Translation of the interview questionnaire. (DOCX 17 kb)
