# Supplementary material for: Determinants of favorable or unfavorable opinion about euthanasia in a sample of French cancer patients receiving palliative care
Source: BMC Palliat Care. 2018 Aug 29;17:104. doi: 10.1186/s12904-018-0357-6 (PMC6114533; doi:10.1186/s12904-018-0357-6)
Supplement: Supplementary file 1 — Translation of the interview questionnaire. (DOCX 17 kb) [file 12904_2018_357_MOESM1_ESM.docx]

Additional file 1: Translation of the interview questionnaire

- Do you believe in God?
  - YES
  - NO
- What is your actual pain in a scale go from 0 to 10
  - 0-10
- Euthanasia is defined by “a doctor intentionally killing a person by the administration of drugs, at that person's voluntary and competent request, to end a situation judged unbearable”.
  Are you favourable to a law permitting euthanasia in France?
  - YES
  - NO
- Deep and continuous sedation is defined by: “sedative and analgesic treatment leading to a profound and continuous change of vigilance to death if the patient is likely to suffer pain, associated with the cessation of all life-sustaining treatments such as artificial nutrition and hydration”. Are you favourable to deep and continuous sedation in terminally phase…?
  - For a patient with a serious and incurable disease whose prognosis is short and has a refractory suffering (suffering not cured by an appropriate treatment)?
    - YES
    - NO
  - For a patient with a serious and incurable disease that decided to stop treatment, when this decision engaged his short prognosis and is likely to cause unbearable suffering?
    - YES
    - NO
  - For a patient unable to express their wish, if the doctor stops life support treatment?
    - YES
    - NO
- Do you think feeding and artificial hydration are:
  - Care ? (all things helping the well-being of the patient, including hygiene, comfort, analgesia and a global care (physic, psychological, social and spiritual) of patient)
  - Treatment ? (all thinks helping to cure, relieve symptoms or prevent a disease occurrence)
- Advances directives are instructions written by an adult in case he would be unable to express his will one day, allowing patients to express their preferences regarding end-of-life care as regards of discontinuation of treatment
  - Do you want advances directives are binding on doctors?
    - YES
    - NO
  - Do you think advances directives have to be subject to a specific duration of validity?
    - YES
    - NO
- Is there any question disturbed you? Have you something to tell about this survey?
